# A bovine respiratory syncytial virus model with high clinical expression in calves with specific passive immunity

**DOI:** 10.1186/s12917-015-0389-6

**Published:** 2015-03-25

**Authors:** Krister Blodörn, Sara Hägglund, Dolores Gavier-Widen, Jean-François Eléouët, Sabine Riffault, John Pringle, Geraldine Taylor, Jean François Valarcher

**Affiliations:** Department of Clinical Sciences, Swedish University of Agricultural Sciences, Host Pathogen Interaction Group, Uppsala, Sweden; Department of Pathology and Wildlife Diseases, National Veterinary Institute, Uppsala, Sweden; Department of Biomedical Sciences and Veterinary Public Health, Swedish University of Agricultural Sciences, Uppsala, Sweden; INRA, Unité de Virologie et Immunologie Moléculaires, Jouy-en-Josas, France; The Pirbright Institute, Pirbright, Surrey, UK; Department of Virology, National Veterinary Institute, Immunology, and Parasitology, Uppsala, Sweden

**Keywords:** Bovine respiratory syncytial virus, Experimental infection model, Calves, Maternal immunity, Aerosol

## Abstract

**Background:**

Bovine respiratory syncytial virus (BRSV) is a major cause of respiratory disease in cattle worldwide. Calves are particularly affected, even with low to moderate levels of BRSV-specific maternally derived antibodies (MDA). Available BRSV vaccines have suboptimal efficacy in calves with MDA, and published infection models in this target group are lacking in clinical expression. Here, we refine and characterize such a model.

**Results:**

In a first experiment, 2 groups of 3 calves with low levels of MDA were experimentally inoculated by inhalation of aerosolized BRSV, either: the Snook strain, passaged in gnotobiotic calves (BRSV-Snk), or isolate no. 9402022 Denmark, passaged in cell culture (BRSV-Dk). All calves developed clinical signs of respiratory disease and shed high titers of virus, but BRSV-Snk induced more severe disease, which was then reproduced in a second experiment in 5 calves with moderate levels of MDA. These 5 calves shed high titers of virus and developed severe clinical signs of disease and extensive macroscopic lung lesions (mean+/−SD, 48.3+/−12.0% of lung), with a pulmonary influx of inflammatory cells, characterized by interferon gamma secretion and a marked effect on lung function.

**Conclusions:**

We present a BRSV-infection model, with consistently high clinical expression in young calves with low to moderate levels of BRSV-specific MDA, that may prove useful in studies into disease pathogenesis, or evaluations of vaccines and antivirals. Additionally, refined tools to assess the outcome of BRSV infection are described, including passive measurement of lung function and a refined system to score clinical signs of disease. Using this cognate host calf model might also provide answers to elusive questions about human RSV (HRSV), a major cause of morbidity in children worldwide.

## Background

Bovine respiratory syncytial virus (BRSV), a pneumovirus in the family *Paramyxoviridae*, is highly prevalent in cattle, with a significant economic impact as the most important viral cause of bovine respiratory disease (BRD) worldwide [1]. Despite the high seropositivity, BRSV outbreaks occur frequently, peaking during the winter months in temperate climates [2]. BRSV is thought to be transmitted by direct and indirect routes, and possibly by aerosol over short distances [3], but all the mechanisms of introduction and maintenance within herds are not clear.

Severe disease is usually observed in calves less than 1 year old, and in particular between 1–3 months in BRSV-endemic regions [4]. BRSV replication in the upper and lower airways causes cellular damage and dysfunction, and may lead to misdirected immune responses, which compound clinical signs of disease [5,6].

Most colostrum fed calves in endemic areas have BRSV-specific maternally derived antibodies (MDA) in serum, affording them limited protection from BRSV infection during the first weeks of life, but having a negative effect on the degree and duration of protection induced by vaccination [7]. The use of commercial vaccines in these animals has not always been fully satisfactory, and the development of a safe and effective BRSV vaccine, with a long duration of protection, therefore remains a high priority for the cattle industry [1]. Furthermore, following vaccination, exacerbated reaction to natural or experimental infection, although uncommon, has been described in calves [8,9], and resembles that previously observed in children immunized with an inactivated vaccine against the genetically and antigenically closely related pneumovirus, human RSV (HRSV) [10].

For these reasons, as well as to improve understanding of the pathogenic mechanisms during an acute infection, a clinically expressive BRSV model is needed to study BRSV pathogenesis, and to evaluate the protective efficacy of vaccine candidates and antivirals.

Several studies have attempted to reproduce field-like BRSV disease in young calves with varying levels of MDA, by administrating BRSV intranasally [11], intratracheally [12-14], or by a combination of intranasal and intratracheal route [14,15]. Some studies report severe clinical disease following experimental BRSV infection, but omit observed or methodological details that would allow interstudy comparison (e.g. rectal temperature [16]). Whereas most studies have failed to reproduce severe clinical signs of disease, despite using high titers of virus and repeated inoculations [17], studies utilizing inoculation by inhalation of aerosol have been those most successful [7,14,18-21], although this is not consistent [22]. Here, our objective was to improve and characterize a BRSV model in calves, by selecting one of two inocula, based on two different strains passaged in calves or in cell culture, and used by two different research groups, to obtain a model that would induce clinical signs comparable to those observed in the field. In addition, we describe a refined scoring system for clinical signs of disease, and objective tools that can be used to monitor and assess the effects of BRSV infection in calves.

## Methods

### Cells and viruses

The BRSV Snook strain was isolated in calf kidney cells [11], and then passaged three consecutive times in gnotobiotic calves by inoculation by respiratory route, and prepared from bronchoalveolar lavage (BAL), as previously described [13] (BRSV-Snk inoculum). BRSV isolate no. 9402022 Denmark [5] was isolated in fetal lung cells, passaged in bovine turbinate cells, and prepared as described previously [21] (passage 8, BRSV-Dk inoculum). Aliquots of the BRSV-Snk and BRSV-Dk inocula were titrated by plaque assay using calf kidney cells, as previously described [11]. Through inoculation of appropriate cell cultures and mycoplasmal or bacterial media, all cells and virus preparations were determined to be free from bovine viral diarrhea virus and bacteria, including mycoplasma (data not shown).

### Animals

The calves included were male, of Swedish Holstein or Swedish red and white breed, and originated from two conventional dairy herds, both free from bovine viral diarrhea virus. The herds were monitored for natural BRSV infections through monthly analysis of BRSV-specific IgG_1_ (see section Detection of BRSV-specific antibodies) in bulk tank milk and in sera from calves, heifers and cows. Herd 1 was monitored from 17 days after the birth of the oldest calf, 1 day after the birth of the second oldest, and before the birth of the remaining calves in study 1. Herd 2, was monitored from 2 months before birth of the oldest calf to be challenged with BRSV in study 2.

For study 1, six calves (A1-3 and B1-3) were obtained from herd 1. These calves had low levels of BRSV-specific serum MDA on the day of challenge; mean 4.1 ± 4.8%COD of kit positive at a dilution of 1:25, where ≤10%COD positive is considered negative by the ELISA kit. In study 2, five calves (C1-5) were obtained from herd 2, all with moderate levels of BRSV-specific serum MDA; mean 49 ± 30% COD positive, or log_10_ titer 2.0 ± 0.2, defined as moderate. In addition, three calves (D1-3) were obtained from herd 1 to act as uninfected controls.

### Challenge and experimental design

Groups of calves were housed in an animal facility, in separate rooms, with free access to clean water and roughage, and additional daily rations of concentrate. Each room had separate negative-pressure ventilation, physical bio-barriers and protective clothing for all staff. All calves were healthy on arrival, and no respiratory clinical signs were observed during one week of acclimation and quarantine. To minimize interference by bacterial co-infections, all calves were treated with antibiotics for five consecutive days (20 mg/kg/day procaine benzyl penicillin intramuscularly). On post-infection day (PID) 0, all calves were challenged by aerosol inhalation.

In study 1, calves were inoculated with either BRSV-Snk (calves A1-3; 9 ± 3 weeks old) or BRSV-Dk (calves B1-3; 9 ± 2 weeks old). Each dose of inoculum contained 10^4.0^ (BRSV-Snk) or 10^4.4^ (BRSV-Dk) pfu of BRSV, diluted in Dulbecco’s modified Eagle medium (DMEM) to a final volume of 5 ml, and aerosolized using a compressor/nebulizer system (Super Dandy Inhaler, PARI, Germany), producing 67% of droplets with a diameter <5 μm, according to the manufacturer.

Inhalation was facilitated by a face mask designed for drug-inhalation in foals (Swevet Piab AB, Sweden). Following challenge, calves were clinically monitored and samples collected until PID 7, when they were euthanized.

In study 2, five calves (C1-5) were challenged with BRSV-Snk and monitored for seven days before euthanasia on PID 7, using the facilities and protocol described for study 1 (except where otherwise noted). In addition to these five calves (6 ± 3 weeks old), post-mortem (PM) BAL samples were collected and analyzed from three healthy calves (calves D1-3; 13 ± 4 weeks old), to act as controls for BAL samples from BRSV infected animals in study 2.

Euthanization was performed by an overdose of general anesthesia (5 mg/kg ketamine and 15 mg/kg pentobarbital sodium) followed by exsanguination.

Approval for both experiments were retained from the Ethical Committee of the district court of Uppsala, Sweden (Ref. no. C330/11). The ethical endpoint of both experiments, defined as the condition when animals would be euthanized prematurely, included: i) marked abdominal dyspnea or respiratory rate >100/min, in conjunction with severely depressed general state, or ii) anorexia for >24 h, or iii) rectal temperature >41°C for >36 h.

### Clinical and pathological examination

Following challenge, daily clinical examinations were performed on each calf, and numerical values were determined for a set of predetermined parameters reflecting general state and respiratory disease (Table 1). Daily individual clinical scores were calculated by summing these numerical values multiplied by a coefficient for each parameter (Table 1). Coefficient weights reflect parameter association with disease severity in BRSV-infected calves less than 3 months of age, based on observations during natural BRSV-outbreaks [23]. Thus, general depression and reduced or absent appetite in BRSV-infected calves were considered moderate to severe signs of BRSV disease with high clinical impact and poor prognosis (coefficients of 4), abdominal dyspnea a moderate sign (coefficient of 3), and increased rectal temperature and respiratory rate, mild to moderate signs (coefficients of 2). The other recorded parameters have varying clinical specificity and severity, from mild to severe, but typically have little clinical impact, and may be very transient. These parameters were assigned a coefficient of 1 (Table 1). Individual accumulated clinical scores (ACS) were calculated as the area under daily clinical scores, using the Trapezoid method. At PM examination, lung lesions were evaluated, recorded and quantified, as previously described [24]. Tissue samples, preferentially from lesioned areas, were collected from each of the lobes in the right lung and trachea, and preserved in 5% paraformaldehyde.

**Table 1:** **Parameters and coefficients used to calculate clinical scores in BRSV infected calves**

**Clinical parameter**	**Parameter coefficient**	**State description**	**Numerical value**
General state	4	Normal	0
		Moves slowly, head down	1
		Lying down/staggers	2
		Recumbent	3
Appetite	4	Normal	0
		Reduced	1
		Absent	2
Abdominal dyspnea	3	Normal	0
		Slight (short, rapid)	1
		Moderate (labored)	2
		Severe (very labored, grunting)	3
Rectal temperature	2	<39.6°C	0
		<40.0°C	1
		<40.5°C	2
		<41.0°C	3
		≥41.0°C	4
Respiratory rate	2	<50/min	0
		<55/min	1
		<65/min	2
		<75/min	3
		≥75/min	4
Intensity of lung sounds	1	Normal	0
		Slightly enhanced	1
		Moderately enhanced	2
		Severely enhanced	3
Added respiratory sounds (wheezing or crackles)	1	Normal	0
		Slight	1
		Moderate	2
		Severe	3
Coughing	1	Absent	0
		Only provoked	1
		Spontaneous, infrequent	2
		Persistent	3
Nasal discharge	1	Normal	0
		Slight uni-/bilat. serous	1
		Moderate bilat. serous to purulent	2
		Copious bilat. purulent	3

During clinical examination, each clinical parameter was assigned a numerical value according to the appropriate state description for that parameter. A clinical score sum was then calculated, by multiplying each numerical value with the parameter coefficient.

### Sampling

Serum was obtained from blood collected on PID −37, −15, 0 and 7, and stored at −20°C, until antibody analysis. Nasal secretions were collected and stored at −70°C, as previously described [21] using sterile cotton-tipped swabs daily from PID 0 to 7, and tampons on PID 0 and 6.

In study 1 endoscopic BAL in sedated calves was performed the day before challenge in all calves as previously described, including disinfection of the endoscope between each calf [23], except lungs were flushed with PBS with 120 μg/ml benzyl penicillin sodium. In both study 1 and study 2 PM BAL was performed in all calves as previously described [12], except lungs were flushed using PBS. BAL fluid was stored on ice after recovery. BAL cells in 10 ml BAL fluid were pelleted by centrifugation (200 × g, 10 min), and resuspended in either 350 μl RLT buffer (Qiagen, Sweden) or 1 ml DMEM with 20% fetal calf serum, and stored at −70°C. BAL supernatant was recovered from centrifugation and stored at −70°C. Bacterial culture was attempted by inoculating bovine blood agar plates with 1 ml of unprocessed BAL fluid.

### Detection of BRSV-specific antibodies

BRSV-specific IgG_1_ antibodies were analyzed using a commercial ELISA kit (SVANOVIR® BRSV-Ab ELISA, Svanova, Sweden), in accordance with the manufacturer’s instructions, including calculations of corrected optic density (COD) and percent of kit positive control (%COD positive).

### Detection and isolation of virus

BRSV-F gene RNA present in nasal secretions or in BAL cells corresponding to 10 ml of BAL, was quantified by RT-qPCR as previously described [21], and expressed as TCID_50_ equivalent units to dilutions of a virus sample with known titer. Accumulated virus shed (AVS) was calculated as the area under individual curves of BRSV detected by RT-qPCR in nasal secretions from PID 0 to PID 7. Virus isolation was attempted by inoculating bovine turbinate cells with BAL and nasal secretion samples, as previously described [21]. Cultures of inoculated bovine turbinate cells were examined daily, and were considered positive if cytopathic effects appeared within seven days.

### Histological analysis

Lung and trachea tissue samples were fixed in 10% buffered formalin, embedded in paraffin, sectioned and stained with hematoxylin and eosin (HE) and by immunohistochemistry (IHC) to detect BRSV antigen.

### BRSV immunohistochemistry staining

For unmasking, sections were treated with heat-induced epitope retrieval (HIER). They were placed in HIER buffer (Target Retrieval Solution, pH = 6, DAKO, Sweden) and subjected to heat treatment in HIER Microwave at 750 W for 7 minutes followed by 350 W for 14 minutes and were allowed to stand for 20 min at room temperature. Endogenous peroxidase activity was blocked with 3% hydrogen peroxide for 20 min at room temperature. Unspecific antigen staining was blocked with 2% bovine serum albumin (Sigma-Aldrich, Sweden AB) for 20 min. The slides were then incubated at room temperature for 45 min with mouse monoclonal antibody anti RSV (clone 5H5, 2G122, 5A6 and 1C3, NCL-RSV3, Novocastra, Leica Microsystems, Sweden) diluted 1:100 in diluents buffer (1% BSA/TBS pH = 7.6). The detection was conducted with the dextran polymer method (EnVisionTM/mouse, DAKO, Sweden). The color was developed with diaminobenzidine substrate (DAB, DAKO, Sweden). Sections were counterstained with haematoxylin. Antibody-omission stained sections served as negative controls for each section. Appropriate positive and negative control sections were included in each run.

### Scoring of histopathological severity of inflammation

The severity of histopathology was scored in each HE-stained section, from 0 (normal), 1 (mild), 2 (moderate) to 3 (severe). The extent and localization of BRSV-antigen was evaluated in IHC-stained sections. BAL cell type composition was determined by manual microscopic analysis of stained cytospin preparations of BAL fluid.

### Detection of cytokines in BAL supernatant

To enhance the sensitivity of cytokine detection, BAL supernatant was concentrated 20X (BAL20X) by filtered (UFC900324, Amicon Ultra-15, 3 kDa, Merck Millipore, Sweden) centrifugation (swinging bucket rotor, 4000 × g, 25–30 min), to an equal final volume. Cytokines in BAL20X were analyzed using commercially available ELISA kits, and by following provided instructions for: interleukin 4 (IL-4; MCA5892KZZ Bovine Interleukin-4 ELISA, BioRad, Sweden), interleukin 6 (IL-6; ESS0029 Bovine IL-6 ELISA, Pierce, USA), interleukin 8 (IL-8; ABIN414016 Bovine IL-8 ELISA, Antibodies Online, Germany), tumor necrosis factor alpha (TNFα; VS0285B-002 Bovine TNFα ELISA, Divbio Science Europe, The Netherlands), and interferon gamma (IFNγ; MCA5638KZZ Bovine IFNγ ELISA, BioRad, Sweden). Cytokine concentration (ng/ml) in each sample of BAL supernatant was calculated using serial dilutions of supplied standards in each kit, and by correcting for the concentration factor of the BAL20X.

### Measuring lung function

Lung function was passively measured before and after BRSV challenge, on PID 0 and 6, by the forced oscillation technique (EquineOsc Calf measurement head, EEMS, Harts, UK), using the same face mask described for aerosol inhalation. Values for resistance (R) and reactance (X)(kPa/L/s) were obtained at 3, 5, 7 and 10 Hz, as described by Reinhold and colleagues [25]. Each calf was tested at least twice on each day and the data sets with optimal coherence selected (coherence > 0.9; majority of data sets > 0.97). In the event of clear artifacts of breathing, such as cough or breath holding, the series were repeated. Daily calibration was performed using a 2.26 m long tube, with a 21 mm internal diameter.

### Ranking of infected calves

To encompass the three major aspects of BRSV-infection clinical signs, lung pathology and virus replication, the six calves in study 1 (calves A1-3 and B1-3) were ranked from least affected (1) to most affected (6) based on: accumulated clinical scores recorded from PID 0 to PID 7; degree of consolidative lesions in lungs on PID 7; and accumulated virus detected in nasal secretions from PID 0 to PID 7. Group rank sums were then calculated for each rank, and for all three ranks (total rank sum).

### Statistical analysis

Where not otherwise stated, results are presented as group mean ± standard deviation (SD). For results presented as a percentage of a whole, SD is presented in percentage points (pp). Statistically significant differences were determined using either one-way ANOVA followed by Student’s *t*-test, or pairwise *t*-test, or Kruskal–Wallis analysis followed by Wilcoxon test (JMP 10 for Mac, SAS Institute Inc.). Significance was assumed when p ≤ 0.05 and tendency when p ≤ 0.1.

## Results

### Study 1: Evaluation of clinical, pathological and virological expression of two virulent BRSV inocula in calves with low levels of MDA

#### Clinical signs following challenge

**Figure 1:**
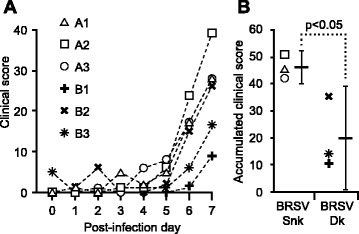
**Daily and accumulated clinical score following aerosol challenge with either BRSV-Snk or BRSV-Dk.** Six calves were experimentally infected with virulent BRSV, either passaged in vivo (BRSV-Snk, n = 3, calves A1-3), or in vitro (BRSV-Dk, n = 3, calves B1-3). Following infection on post-infection day (PID) 0, calves were monitored for seven days. Daily clinical scores (panel **A**) were calculated from observed clinical signs (see Tables 1 and 2). Accumulated clinical scores, from PID 0 to PID 7 (panel **B**), were calculated as the area under individual clinical score curves.

Following experimental infection, mild to severe clinical signs of respiratory disease were observed in all infected calves (Figure 1A). For all calves, upper respiratory signs, such as nasal discharge and coughing, as well as ocular discharge were observed on PID 3–5. In BRSV-Snk infected calves, these progressed to severe respiratory signs on PID7, whereas clinical signs were more moderate on PID 7 in calves infected with BRSV-Dk (Table 2).

**Table 2:** **Clinical scores in calves on day seven after experimental infection with BRSV**

	**Group/Calf**					
	**BRSV-Snk**			**BRSV-Dk**		
**Clinical sign**	A1	A2	A3	B1	B2	B3
General state	1	1	1	0	1	0
Appetite	1	1	1	0	0	0
Abdominal dyspnea	2	2	2	2	2	2
Temperature	0	4	0	0	1	2
Respiratory rate	3	3	3	1	3	0
Lung sounds intensity	2	3	3	0	2	1
Added lung sounds	0	3	2	0	2	1
Nasal discharge	1	2	1	1	1	1
Coughing	2	2	2	0	2	0
**Clinical score**	25	38	28	9	25	13

Six calves were experimentally infected with virulent BRSV, either passaged in vivo (BRSV-Snk, n = 3, calves A1-3), or in vitro (BRSV-Dk, n = 3, calves B1-3). Clinical signs were recorded daily for seven days, and scores calculated as described in Table 1.

Consequently, compared to BRSV-Dk infected calves, BRSV-Snk infected calves had significantly higher (p ≤ 0.05) accumulated clinical scores (Figure 1B).

### Macroscopic and histological lung pathology

**Figure 2:**
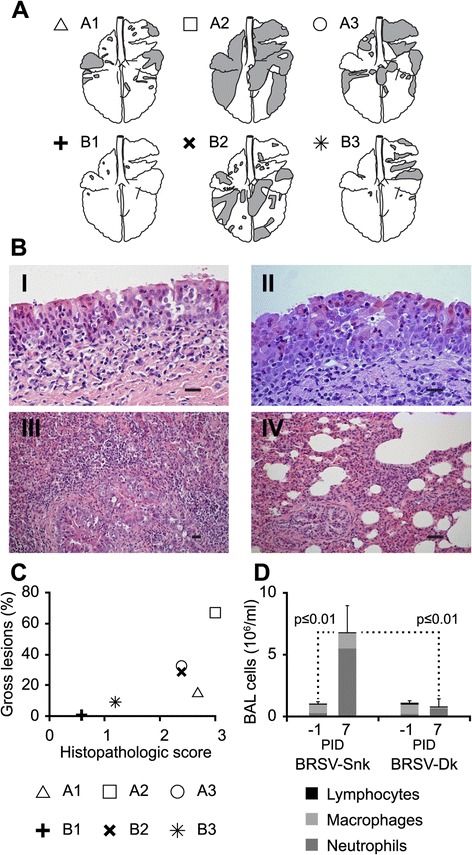
**Pulmonary pathology and neutrophil influx in calves following aerosol challenge with either BRSV-Snk or BRSV-Dk.** Calves were experimentally infected as described in Figure 1. Calves were euthanized seven days after infection and the macroscopic extent of lung lesions were documented (panel **A**). From each calf, trachea tissue and four lung tissue samples were collected for sectioning, staining and histopathological description and scoring of severity of inflammation (1–3). Panels **B**:I-IV show representative HE-stained sections from: (**B**:I) trachea from calf A2; (**B**:II) trachea from calf B3; (**B**:III) lung from calf A2; and (**B**:IV) lung from calf B3. Horizontal bars indicate 50 μm in panels **B**:I and **B**:II, and 100 μm in panels **B**:III and **B**:IV. Mean histopathological severity of inflammation per calf, is shown on the x-axis in panel **C**, along with the proportion (%) of macroscopic lung lesions per calf on the y-axis. Bronchoalveolar lavage (BAL) was performed on PID −1 and PID 7, and cell types in BAL samples enumerated (Panel **D**). Stacks represent the mean total number of cells in BAL per ml, with associated standard deviation, as well as the number of neutrophils, macrophages and lymphocytes in BAL per ml. The proportion of eosinophils were <1% in all samples.

BRSV-Snk infected calves tended to have more extensive consolidated lung lesions (38.5 ± 26.3% of total lung tissue) on PID7, compared to calves infected with BRSV-Dk (12.8 ± 14.6%), but this difference was not statistically significant (p = 0.23; Figure 2A and C).

Histologically, lesions in the trachea in both groups of calves consisted of degeneration and necrosis of epithelium, and epithelial hyperplasia in some areas (Figure 2B:I and II show representative pictures from BRSV-Snk and BRSV-Dk infected animals, respectively). In the lungs, BRSV-Snk infected calves showed extensive moderate to severe bronchointerstitial pneumonia, as well as purulent bronchitis and bronchiolitis (Figure 2B:III; representative picture of lung, calf A2). BRSV-Dk infected calves showed similar but less severe histopathological changes in the lungs, ranging from mild to moderate (Figure 2B:IV; representative picture of lung, calf B3).

In summary, BRSV-Snk infected animals tended to have macroscopically more extensive, and histologically more severe lung lesions, compared to BRSV-Dk infected animals (Figure 2C), but with no discernible difference in the severity of histological inflammation in the trachea.

### Inflammatory cells in bronchoalveolar lavage

Bronchoalveolar lavage was performed to collect BAL cells in all calves, once before infection (PID −1), and again on the day of euthanization (PID 7). Regardless of challenge inoculum, experimental infection altered the composition of BAL cell types (Figure 2D). Before infection, the predominant BAL cell type were macrophages (63.0 ± 26.0% for BRSV-Snk and 69.0 ± 10.1% for BRSV-Dk), followed by neutrophils (30.0 ± 27.8% for BRSV-Snk and 21.3 ± 18.5% for BRSV-Dk), whereas after infection, neutrophils were the predominant BAL cell type (79.0 ± 4.4% for BRSV-Snk and 80.3 ± 6.0% for BRSV-Dk), followed by macrophages (18.7 ± 2.3% for BRSV-Snk and 17.7 ± 4.5% for BRSV-Dk) (Figure 2D).

However, the total number of BAL cells was significantly increased only in BRSV-Snk infected calves following challenge (6.9 ± 2.0 ×10^6^ cells/ml at PID 7), compared to before challenge (1.0 ± 0.2 ×10^6^ cells/ml at PID −1; p ≤ 0.01; pairwise *t*-test), and compared to BRSV-Dk infected calves before and after challenge (1.1 ± 0.2 ×10^6^ cells/ml at PID −1; 0.8 ± 0.7 ×10^6^ cells/ml at PID 7; p ≤ 0.01; pairwise *t*-test; Figure 2D).

### Virology

#### RT-qPCR detection and isolation of BRSV in nasal secretion and BAL

**Figure 3:**
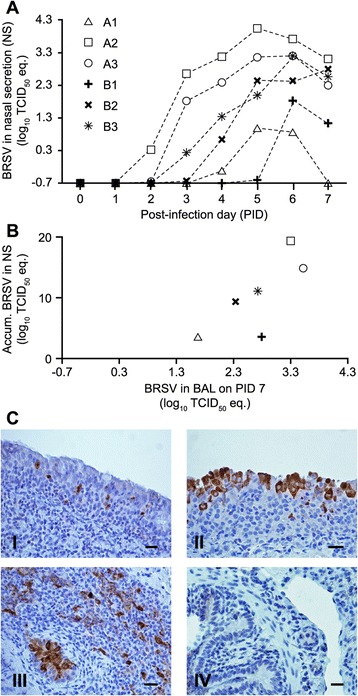
**Virus detected in the airways of calves after aerosol challenge with either BRSV-Snk or BRSV-Dk.** Calves were experimentally infected as described in Figure 1. Daily nasal secretion (NS) samples were collected for eight consecutive days, starting on PID 0. After euthanization on PID 7, bronchoalveolar lavage (BAL) was collected, along with tissue samples from the trachea and lung, for histopathology and immunohistochemistry (IHC) to demonstrate BRSV-antigen (brown stain). BRSV RNA in daily NS (panel **A**) and BAL (x-axis, panel **B**) was detected by RT-qPCR, and is expressed as log_10_ TCID_50_ equivalent unit. The accumulated virus shed in NS (y-axis, panel **B**) was calculated as the area under individual curves. Panels **C**:I-IV show representative IHC-stained sections of: (C:I) trachea from calf A2; (C:II) trachea from calf B3; (C:III) lung from calf A2; and (C:IV) lung from calf B2. Horizontal bars in panels C:I-IV indicate 50 μm.

BRSV RNA was detected by RT-qPCR in nasal secretions collected daily from PID 0 to PID 7, and in BAL collected on PID 7 (PM BAL). In addition, BRSV was isolated in the first passage in bovine turbinate cell culture, from all infected calves, in both nasal secretions from PID 6, and PM BAL fluid. Attempted bacterial culture from BAL fluid indicated no bacterial coinfection in the lungs of any of the calves. Two of the BRSV-Snk infected calves (A2 and A3) started shedding virus on PID 2, and shed high amounts of virus (A2 log_10_ AVS 19.3 TCID_50_ equiv.; A3 log_10_ AVS 14.9 TCID_50_ equiv.), both in nasal secretions and PM BAL, whereas the third BRSV-Snk infected calf (A1), shed substantially less virus (log_10_ AVS 3.6 TCID_50_ equiv.) (Figure 3A-B).

Compared to the two high-shedding BRSV-Snk infected calves, calves infected with BRSV-Dk shed markedly less virus in nasal secretions (B1, B2 and B3 log_10_ AVS 3.5, 9.4 and 11.1 TCID_50_ equiv., respectively), and had less viral RNA in BAL, although this was not statistically significant (Figure 3A-B).

### BRSV immunostaining in lung and trachea sections

In IHC-stained sections of trachea very little or no BRSV antigen was detected in BRSV-Snk infected calves (Figure 3C:I is representative), whereas viral antigen was abundant in sections of trachea from BRSV-Dk infected calves (Figure 3C:II is representative). Conversely, whereas viral antigen was abundant in the lungs of 2/3 BRSV-Snk infected calves (Figure 3C:III is representative), very little or no BRSV antigen was detected in the lungs from BRSV-Dk infected calves (Figure 3C:IV is representative). The third BRSV-Snk infected calf (A1) was negative for BRSV antigen by IHC, both in the trachea and in the lungs (data not shown).

### Serum BRSV-specific antibodies

**Figure 4:**
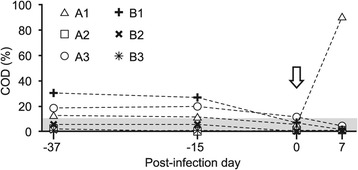
**Serum anti-BRSV IgG**_**1**_ **in calves, before and after aerosol challenge with either BRSV-Snk or BRSV-Dk.** Calves were experimentally infected as described in Figure 1. BRSV-specific IgG_1_ antibodies, detected by ELISA (SVANOVIR® BRSV-Ab ELISA, Boehringer Ingelheim Svanova, Sweden) in serum diluted 1:25, are expressed as percent of the corrected optical density (COD) of a positive control sample. The shaded area of the chart indicates ≤10% COD of positive, defined as negative by the kit manufacturer.

All calves, except A1, had low and consistently decreasing levels of BRSV-specific MDA, throughout the experiment (Figure 4). In contrast, the BRSV-Snk infected calf A1 (the oldest calf in study 1) seroconverted within 7 days after challenge, strongly suggesting that this calf had been previously primed against BRSV.

### Animal ranking

**Figure 5:**
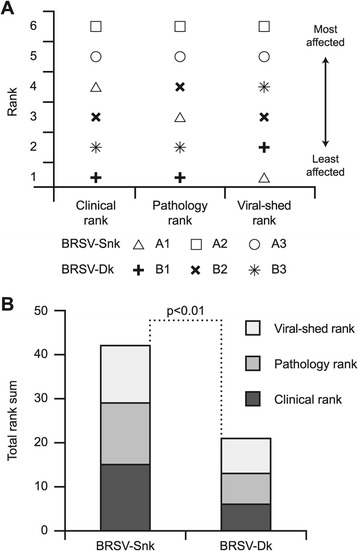
**Clinical, pathological and virological ranking of calves following aerosol challenge with either BRSV-Snk or BRSV-Dk.** Calves were experimentally infected as described in Figure 1. Following challenge, calves were ranked (panel **A**) based on accumulated daily clinical scores (Clinical rank), nasal virus shed (Viral-shed rank), and extent of lung lesions (Pathology rank). Panel **B** shows the rank sum for each of the three ranks, and the total rank sum per group.

When calves were ranked from least affected (1) to most affected (6) based on clinical score, degree of lung pathology and accumulated virus shed in nasal secretions, two of the BRSV-Snk infected calves (A2 and A3) consistently received the highest ranks (Figure 5A). Conversely, the calves infected with BRSV-Dk received low ranks, as they demonstrated less severe clinical signs, less lung pathology, and less virus shedding (Figure 5A). The BRSV-Snk infected calf that rapidly seroconverted following challenge (A1), received a high clinical rank, an intermediate lung pathology rank, and a low viral-shed rank (Figure 5A). Overall, the BRSV-Snk infected calves ranked significantly higher, compared to calves infected with BRSV-Dk (p ≤ 0.01; Figure 5B). Based on the overall ability of the BRSV-Snk inoculum to induce BRSV infection, it was chosen as the inoculum in study 2, to reproduce and characterize the model in calves with moderate levels of MDA.

### Study 2: Reproduction of clinical signs, virology and pathology using aerosolized BRSV-Snk in calves with passive immunity

Based on the high level of clinical signs of disease observed in calves with low levels of MDA, following challenge with BRSV-Snk in study 1, an additional five calves (calves C1-5), which were all BRSV-naive and had moderate levels of BRSV-specific serum IgG_1_ MDA were challenged using the same inoculum and protocol as used in study 1.

### Clinical signs, lung pathology and virology following challenge

**Figure 6:**
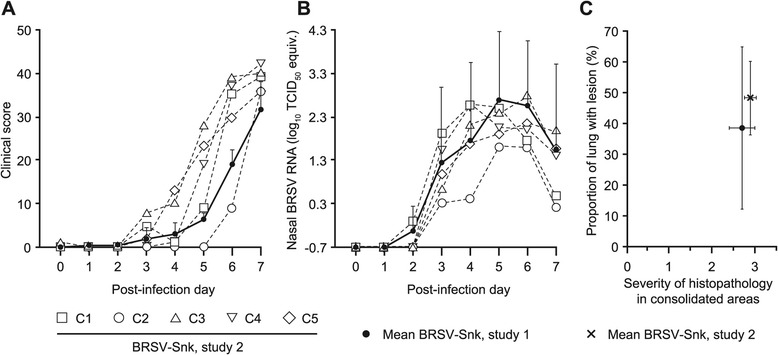
**BRSV-Snk challenge in calves with low or moderate passive immunity: clinical, virological and pathological outcomes.** Experimental challenge by inhalation of aerosolized BRSV passaged in gnotobiotic calves (BRSV-Snk) was performed in three calves (A1-3; study 1) with low levels of BRSV-specific maternal antibodies (MDA), and later reproduced in five calves (C1-5; study 2) with moderate levels of MDA. Clinical scores (Panel **A**) and BRSV RNA detected by RT-qPCR in nasal swab samples (Panel **B**), from the day of challenge (post-infection day or PID) 0 to PID 7. Individual values are presented for calves C1-5, and mean values are presented for calves A1-3. For both groups of calves, panel **C** shows mean extent of macroscopic lung lesions on the y-axis, expressed as a percent of total lung area, and the mean severity of histopathological inflammation, scored from 0 to 3, on the x-axis. For mean values, vertical and horizontal lines indicate standard deviation.

Clinical signs of disease and lung pathology, as well as levels of viral RNA detected in the upper and lower airways in study 2, following challenge of calves C1-5, have been described in detail elsewhere [24]. The amplitude and kinetics of these parameters were in line with observations in BRSV-Snk infected calves in study 1 (Figure 6A-C). Briefly, all infected calves in study 2 developed clinical signs of upper respiratory disease starting on PID 3–5, which progressed to severe lower respiratory disease from PID 5 to PID 7 (Figure 6A). On PID 7, all five calves were moderately to severely depressed (recumbent, and staggering when prompted to rise), with reduced or absent appetite. Although both groups of BRSV-Snk infected calves in study 1 and 2 shed high amounts of virus, as detected by RT-qPCR, calves in study 2 shed less accumulated virus in nasal secretions compared to those in study 1 (log_10_ 1.6 TCID_50_ eq. difference in mean), but more in BAL fluid on PID 7 (log_10_ 2.1 TCID_50_ eq. difference in mean). At post-mortem, BRSV-Snk infected calves in study 2 had extensive consolidated lung lesions and histopathological changes on PID 7, similar in extent to those in study 1 (38.5 ± 26.3% and 48.3 ± 12.0% of total lung area for study 1 and study 2, respectively; Figure 6C; mean histological score 2.7 ± 0.3 and 2.9 ± 0.1 for study 1 and study 2, respectively; Figure 6C).

### Quantitative assessment of lung function

**Figure 7:**
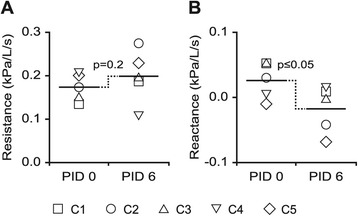
**BRSV-Snk challenge in calves with low or moderate passive immunity: effect on airway resistance and reactance.** Experimental challenge by inhalation of aerosolized BRSV passaged in gnotobiotic calves (BRSV-Snk) was performed in five calves with moderate levels of BRSV-specific maternal antibodies (MDA). Lung function was measured using the forced oscillation technique, and a tightly fitting face mask; before challenge on post-infection day (PID) 0, and after challenge, on PID 6. Resistance (Panel **A**) and reactance (Panel **B**) at 10Hz were calculated and presented as kPa/L/s.

The impact of lower respiratory disease (as demonstrated by clinical signs of disease and lung pathology) on lung function in the five calves in study 2 was evaluated by the forced oscillation technique before and after challenge (on PID 0 and 6). Following challenge, infected animals demonstrated a tendency at 10 Hz measurements for increased airway resistance (0.17 ± 0.03 kPa/L/s on PID 0; 0.20 ± 0.06 kPa/L/s on PID 6; p = 0.2, pairwise *t*-test) and significantly decreased airway reactance (0.03 ± 0.03 kPa/L/s on PID 0; −0.02 ± 0.04 kPa/L/s on PID 6; p ≤ 0.05, pairwise *t*-test; Figure 7).

### Cytology and cytokine profile in BAL

**Figure 8:**
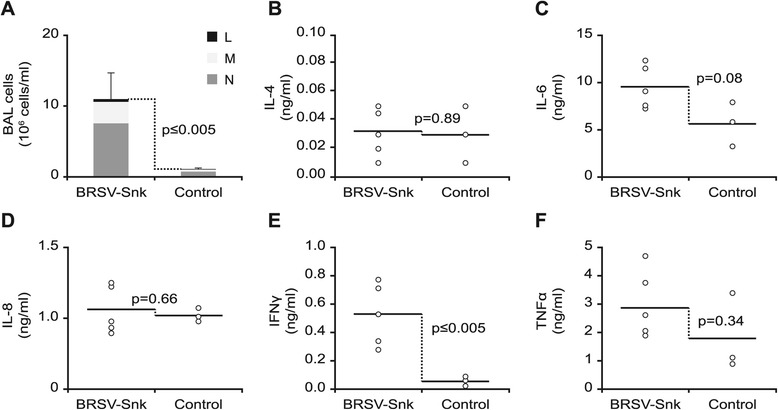
**Variations of cells populations and cytokines in bronchoalveolar lavage (BAL) from calves challenged with BRSV-Snk.** Experimental challenge by inhalation of aerosolized BRSV passaged in gnotobiotic calves (BRSV-Snk) was performed in five calves with moderate levels of BRSV-specific maternal antibodies (MDA). Samples of the cells populating the lower airways were collected via BAL seven days after infection, and in addition, from three healthy uninfected calves (Control). Cells in BAL samples were analyzed by light microscopy (panel **A**), and expressed as group mean ×10^6^ cells/ml. The concentrations of indicated inflammatory cytokines in BAL sample supernatants were measured using specific ELISAs, and are expressed in ng/ml: (panel **B**) interleukin (IL)-4; (panel **C**) IL-6; (panel **D**) IL-8; (panel **E**) interferon gamma (IFNγ); and (panel **F**) tumor necrosis factor alpha (TNFα). Individual data are indicated by rings and group means by horizontal lines. Probability (p) of statistically significant differences between groups is given in each panel, where p ≤ 0.05 was considered statistically significant.

Seven days after experimental BRSV infection, BAL was collected from all five infected calves in study 2, and in addition, from three uninfected calves. BAL cell types in cytospin preparations were analyzed by light microscopy (Figure 8A) and BAL supernatant was analyzed using ELISAs, specific to bovine inflammatory cytokines (Figure 8B-F).

Following infection, infected calves demonstrated a significant increase in inflammatory cells in BAL (11.0 ± 3.7 ×10^6^ cells/ml), compared to uninfected controls (1.1 ± 0.2 ×10^6^ cells/ml) (p ≤ 0.005; Figure 8A). As observed in BRSV-Snk infected calves in study 1, this increase consisted mostly of neutrophils (68.6 ± 14.4%), followed by macrophages (27.8 ± 13.0%) and lymphocytes (3.6 ± 2.0%). Very few eosinophils were seen in the BAL of infected calves (<0.5%).

Cytokine analysis of BAL supernatant from infected calves demonstrated significantly higher levels of IFNγ (p ≤ 0.005; Figure 8E) and a tendency for higher levels of IL-6 (p = 0.08; Figure 8C), compared to uninfected control calves. In contrast, levels of IL-4, IL-8 and TNFα in BAL, did not differ from those of uninfected controls, seven days after infection (Figure 8B,D and F).

## Discussion

In the present paper, we describe an experimental model of BRSV infection with strong clinical and pathological expression in calves with maternal antibodies. This model combines and refines elements from previously published studies, including aerosol inoculation, the use of inoculum passaged in gnotobiotic calves, and methods to monitor and quantify clinical, pathological and virological parameters [12,14,21]. We believe that this model can serve to enable a better evaluation of vaccine and antiviral safety and efficacy and further increase understanding of the pathogenesis of BRSV, and also of HRSV.

Regardless of the inoculum, all inoculated calves (n = 11), in both studies, developed manifest BRSV disease. The rapid seroconversion detected in calf A1 in study 1 indicated that, in contrast to the other calves, this BRSV-Snk-infected calf had been previously exposed to natural BRSV. This highlights that, to ascertain BRSV naiveté by seromonitoring in herds, seronegative sentinel animals need to be regularly monitored during the entire lifespan of calves to be included in experimental trials. This case also confirms earlier reports [26,27] that a sufficient amount of MDA can suppress detectable humoral immune responses, following BRSV infection in young calves, with the net effect of declining MDA detected by ELISA. However, although this previous priming appear to have provided some virological protection, compared to all other BRSV-Snk infected calves, calf A1 demonstrated severe clinical disease following BRSV infection, in the absence of any other detected pathogen, contrary to previously published reports [28,29]. In contrast, the moderate clinical signs, pathology and virus shed observed in calf B1 following BRSV-Dk challenge, may possibly be explained by favorable genetics, with more efficient innate and cellular responses. Any previous BRSV exposure of calf B1, even if virus replication was very limited, would have resulted in a rapid anamnestic humoral immune response upon reinfection, as seen in calf A1, and demonstrated elsewhere [24,30].

Immunohistochemical staining for BRSV antigen in study 1 showed a marked difference in localization of virus on PID 7, where two BRSV-Snk infected calves had large amounts of virus in the lungs and only small amounts of virus in the trachea, while the reverse was true for BRSV-Dk infected calves. This disparity in antigen localization on PID 7 might be due to delayed progression of viral replication in BRSV-Dk infected calves. This opens the possibility that BRSV-Dk infected calves might have developed more severe clinical signs, if the 7-day challenge model had been abandoned and the experiment had been prolonged. However, this would contradict previous observations using the BRSV-Dk inoculum, which indicate a peak of clinical signs on PID 6 [20]. Nonetheless, virus was isolated and high quantities of viral RNA were detected by RT-qPCR in samples from the upper and lower airways from all infected calves in both studies, including calves A1 and B1, although BRSV-Snk infected calves from both studies shed 10^3^ times more virus in nasal secretions than BRSV-Dk infected calves.

Despite the differing data of calves A1 and B1, and although the number of animals in the first study was low (n = 3 + 3), we concluded that BRSV-Snk infected calves tended to be more severely affected in the 7-day experimental infection model, compared to BRSV-Dk infected calves, when summarizing clinical, pathological and virological parameters (Figure 5B). Thus, results from study 1 were reproduced in study 2, using aerosol inoculation of the BRSV-Snk inoculum in an additional five BRSV-naive calves, with moderate levels of MDA.

Using a minimal amount of aerosolized inoculum (10^4.0^ pfu for BRSV-Snk) to experimentally infect calves with and without MDA, the kinetics of severe naturally occurring BRSV infection in calves was recreated in this study [4,31]. On PID 7, most calves were severely affected, and all BRSV-Snk infected calves in study 2 were demonstrating depression, anorexia, pyrexia, tachypnea, abdominal dyspnea and wheezing lung sounds.

The high level of clinical expression in BRSV-Snk infected calves was mirrored by the great extent of macroscopic lung lesions and by the severity of histopathological changes in the lungs on PID 7. Manifest inflammation was further verified in BRSV-Snk infected calves in study 2, with significantly increased numbers of neutrophils, macrophages and lymphocytes in BAL, similar to that reported following natural BRSV infection in calves [32].

At the peak of clinical signs, 7 days post infection, the calves in study 2 also demonstrated an increase of IFNγ and minimal amounts of IL-4 in BAL supernatant, which agrees with previously reported responses to primary BRSV infection in calves [33]. Previous studies have shown that T lymphocytes migrating to the lung during BRSV infection are predominantly IFNγ producing CD8^+^ T cells [34,35], which have been shown to be important for BRSV clearance [12,36]. However, at least a proportion of the IFNγ detected in BAL supernatant in study 2, may also have been produced by NK cells, or alveolar macrophages, as have been shown in vitro with human alveolar macrophages [37]. Similar to that seen in calves, infants hospitalized with severe HRSV bronchiolitis, had an increased frequency of IFNγ producing CD8^+^ T cells, collected by nasal brush, compared to infants with milder upper respiratory tract infections [38]. Thus, IFNγ in BAL supernatant can serve as an objective measurement of disease severity, following experimental BRSV challenge.

Elevated levels of TNFα, IL-6 and IL-8 in BAL or serum have also been associated with clinical signs and pathology caused by BRSV infection in calves [33,39,40]. The lack of detectable increases in these cytokines in BAL supernatant on PID 7 in study 2 may have been due to suboptimal timing of BAL collection, as another study where 6 weeks old calves where infected with BRSV reported TNFα and IL-6 concentrations in BAL to peak on PID 9 and PID 3, respectively [41].

The patent pneumonia in the BRSV-Snk infected calves following infection in study 2, as demonstrated by clinical signs, lung pathology, and the inflammatory picture in BAL, also reduced the lung function of affected animals; with increased airway resistance, and decreased airway reactance, which is suggestive of bronchoconstriction and obstructive airway processes [25,42]. The objective measurement of lung function by the forced oscillation technique can be a useful tool in further quantifying the outcome of a BRSV challenge, and the efficacy of vaccine candidates, in calves. Optimization of materials (e.g. face mask and tubing) and methods (e.g. frequencies used) could further improve the analysis, and need to be investigated in a larger set of calves.

The relative potency of the BRSV-Snk inoculum might be due to loss of virulence in the BRSV-Dk inoculum, following passage in cell culture. This is supported by previously published studies, which demonstrated a higher level of clinical signs of respiratory disease in calves with MDA, using the same mode of inoculation and the same isolate as the BRSV-Dk inoculum, but with fewer passages in vitro [21], and by propagation in fetal lung cells [20]. Loss of virulence following in vitro passage has been reported in some studies for BRSV and HRSV [43,44], but not in others [45,46], and likely depends on the type of cells and number of passages, and may be associated with alterations in protein expression and post-translational modifications. The BRSV-Snk inoculum, in contrast to the BRSV-Dk inoculum, had been passaged in gnotobiotic calves.

Apart from the inoculum, challenge by aerosol inhalation hinges on two principal factors: the quality of the aerosol, and the quantity inhaled by each animal. Limited experimental infection studies in calves, using similar aerosolization of BRSV in conjunction with intratracheal injection [47], indicate that virus is mainly deposited in the upper airways using this method, with subsequent progression of virus replication to the lower airways. However, other studies using inhaled aerosols show that droplets ≤5 μm in diameter (67% of droplets in the present study) can reach the alveoli in humans [48], and reach the whole lung when infecting steers with aerosolized foot-and-mouth disease virus [49], and more accurately reproduces the symptoms of natural infection, compared to large droplet intranasal administration, when human volunteers were infected with influenza [50]. Thus, more research on the kinetics of natural BRSV infection is needed, to complement experimental findings, and to further elucidate the relevance of the model with regard to BRSV pathogenesis.

To study the unmodified pathogenesis of BRSV, field-like clinical signs are essential, and to calculate relevant treatment effects in vaccine or antiviral trials, a minimum clinical expression is required, making the model presented herein highly relevant, in contrast to comparable models with less clinical signs [51-53], or comparable clinical expression, but less neutralizing MDA at the time of challenge [7]. This cognate host calf model might also provide further understanding about HRSV in infants [54-56], with particular usefulness in the study of RSV pathogenesis and pathological processes in the lower airways, where data from infants is limited [57], but also to evaluate candidate vaccines that utilize proteins conserved across BRSV and HRSV [24].

## Conclusions

In conclusion, we have established a BRSV model with a severe clinical expression in calves with maternal antibodies at the time of challenge. We furthermore describe tools to evaluate disease severity: consistently, using a rigid and comprehensive clinical scoring system; and objectively, using a passive lung function test and IFNγ concentration in BAL, to complement established parameters, such as extent of lung lesions and virus shedding following challenge. These tools can be used in future BRSV research and vaccine development studies and this model could also be valuable for the understanding of HRSV.

## Abbreviations

ACS: Accumulated clinical scores
AVS: Accumulated virus shed
BAL: Bronchoalveolar lavage
BRD: Bovine respiratory disease
BRSV: Bovine respiratory syncytial virus
BRSV-Dk: BRSV, isolate no. 9402022 Denmark
BRSV-Snk: BRSV, Snook strain
COD: Corrected optic density
DMEM: Dulbecco’s modified Eagle medium
ELISA: Enzyme-linked immunosorbent assay
HE: Hematoxylin and eosin
HIER: Heat-induced epitope retrieval
HRSV: Human respiratory syncytial virus
IFNγ: Interferon γ
IHC: Immunohistochemistry
IL-4: Interleukin 4
IL-6: Interleukin 6
IL-8: Interleukin 8
MDA: [BRSV-specific] maternally derived antibodies
PID: Post-infection day
PM: Post-mortem
RNA: Ribonucleic acid
RT-qPCR: Real-time quantitative polymerase chain reaction
SD: Standard deviation
TCID50: 50% tissue culture infective dose
TNFα: Tumor necrosis factor alpha
